# Diver's and "Caisson" Disease

**Published:** 1904-05-07

**Authors:** 


### The Hospital.

A Journal of

The Medical Sciences and Hospital Administration.

Vol. XXXVI.—No. 919. MAY 7, 1904.

### DIVER'S AND "CAISSON" DISEASE.

The announcement by Professor Leonard Hill, F.R.S., of his discovery of the mode of causation of the complicated and obscure symptoms which have for a long time been classed under the general term of "diver's disease," and which have proved a serious impediment to many great works of modern engineering from their liability to occur in men who were working under increased atmospheric pressure in caissons, may justly be regarded as a triumph of scientific research. In the construction of the great bridge built across the Mississippi by Captain Eads in 1870, out of 352 men employed in working under various degrees of pressure, 30 became seriously affected, and no less than 12 of these cases terminated fatally; and although, in the construction of the Blackwall Tunnel, Sir Alexander Binnie found that the dangers of the work could be much diminished by improved ventilation, they were still of a very serious character, and such as to place great difficulties in the way of engineering enterprises requiring caisson work. In diving operations the same difficulties fixed a limit of depth beyond which nothing could be done; and, although a few men of exceptional physique have been able to work at depths reaching even to 180 feet, the ordinary diver has not been able to go much below 100, or to remain long even there. The pathology of the disease has been no less puzzling than the precise method of causation. Some of the sufferers have died speedily, others have become paralysed in different localities, either temporarily or permanently, others have had intense pains and swelling of the joints, and the link between these protean maladies was undiscovered. All that could be described as certain was that an engineer in charge of caisson work was compelled to provide against a large amount of disabling sickness among his men; and that many diving undertakings calculated to be of a highly profitable character were practically prohibited by the impossibility of finding a sufficient number of skilled men who would encounter the grave risks involved.

Like many other discoveries of great practical value, that of Professor Hill is of extreme simplicity, and, as soon as it is announced, serves to bring the scattered and previously unintelligible facts into a coherent whole. By careful experiments upon animals, carried out in conjunction with Professor McLeod, he has found that the blood, when under pressure, dissolves a considerable and constantly-increasing amount of atmospheric gases, and that these tend to escape rapidly when the pressure is removed. The result is a sort of frothing or effervescence; and the exceedingly variable character of the consequent symptoms depends partly upon the rapidity of the effervescence, and partly upon the locality in which it occurs. If it be in any large vein near the heart the effect may be speedily fatal, just as when an animal is designedly killed by blowing air into its jugular vein, or as when air enters the circulation in the course of a surgical operation. If it be in the vicinity of a motor centre, temporary or permanent paralysis may be produced; while all local pains and swellings admit of similar explanation. The whole secret of avoiding danger is to remove the compression so gradually that no effervescence is permitted to occur. For this purpose a special diving bell or caisson for "decompression" is required, and has been designed by Messrs. Siebe and Gorman. With this apparatus still further experiments have been conducted; and Professor Hill believes that it will render diving possible and safe up to depths of 200 feet, beyond which he does not expect to be able to go, on account of the poisonous character of highly compressed oxygen. The easy attainment of such a depth will, however, materially increase the existing range of work, and will not only bring a great amount of sunken treasure, both natural and artificial, within easy range of access, but will also remove an element of danger which has never hitherto been absent from any undertakings for which either diving or caisson work was required. Contractors of a certain type will be likely, of course, to grumble at the time required for safe decompression, and to say that it will add unnecessarily to the cost of their operations; and such persons will probably find labourers who are willing to incur avoidable risks for a consideration. However this may be, it will at least be no longer possible for greed and recklessness to find shelter under the plea of ignorance.

A noteworthy feature of Professor Hill's discovery is that it must have remained in the region of conjecture if it had not been substantiated by a series of experiments upon animals. In no other way could either the occurrence or the precise nature of the blood-frothing have been demonstrated, and in no other way could the safety afforded by slow decompression have been placed beyond the reach of doubt. The event adds yet one more to the long list of benefits conferred upon mankind by the physiologists for whose proceedings a bevy of old women of both sexes can find no words of adequate condemnation; and it may reasonably be cited as a typical example of what such experiments have effected, and will certainly effect again, for the preservation of human life and the diminution of human suffering. On the present occasion it has solved a problem which had completely baffled physicians, and it has rescued important industries from dangers which, in many cases, were such as even to be prohibitory of any extensive attempts to engage in them.

